# Authenticity, Interactivity, and Collaboration in VR Learning Games

**DOI:** 10.3389/frobt.2018.00133

**Published:** 2018-12-19

**Authors:** Meredith M. Thompson, Annie Wang, Dan Roy, Eric Klopfer

**Affiliations:** Comparative Media Studies and Writing, Massachusetts Institute of Technology, Cambridge, MA, United States

**Keywords:** immersive virtual reality, stem education, game based learning, embodied learning, K12 education, collaboration

## Abstract

Decreasing cost and increasing technology access in schools places 3D immersive virtual reality (VR) within the reach of K-12 classrooms (Korbey, 2017). Educators have great interest in incorporating VR into classrooms because they are engaging and often novel experiences. However, long-term curriculum development must be positioned on how to best leverage the unique affordances of VR, be informed by theory and research, and integrate VR in meaningful ways that continue to motivate students even after experiences are no longer novel. We propose the theoretical framework of embodied learning and discuss how VR and reflect on current research findings to outline effective applications of VR and provide guidelines in developing educational materials using those tools. We discuss two particular examples: spatial awareness and collaboration. We share our perspectives on the benefits and challenges of applying these principles in a learning game about cellular biology.

## Background

VR has the potential to broaden the reach of the traditional classroom by addressing limitations of K-12 classroom environments. VR simulations that engage learners as explorers shift the focus from content acquisition to active inquiry (Hew and Cheung, 2010; Merchant et al., 2012; Ahn et al., 2017). Now that these technologies are within reach of classrooms and lecture halls, research needs to move beyond simply asking whether VR can help bolster learning, and consider how best to use these tools in educational contexts (Dalgarno et al., 2011). In doing so, we not only imagine the types of problems that immersive 3D can solve for K-12 educators but consider the larger question of how to craft learning experiences for students that effectively move between and utilize two dimensional, three dimensional, and immersive 3D visualizations.

Our labs have developed a number of learning simulations and games, and are currently developing a game to introduce students to cellular biology. Through this process, we have gained perspective on the benefits and challenges of using VR in creating authentic, interactive, and collaborative experiences that help students learn about the complex topic of cellular biology. First, we explore how VR can be helpful in creating authentic representations in biology. Then, we discuss current understandings of the theoretical frameworks of embodied learning and collaborative learning. Finally, we discuss how we have applied these two perspectives through a collaborative, cross-platform educational game named *Cellverse*.

### Authenticity: Cell Biology as a Context for Virtual Reality

Cells and the central dogma are two critical topics in biology standards and curricular materials (National Research Council, 2013). Despite the importance of these concepts, visuals of cells are often oversimplified in introductory resources (Shi et al., 2010; Tibell and Rundgren, 2010) and misunderstood by students and educators alike (Çeliker, 2013; Vlaardingerbroek et al., 2014). Incorporating 3D visualization such as immersive VR into biology curricula may be a solution to improving student learning. Previous research has shown a notable positive correlation between the use of visual models and student scores—just a few class sessions of exposure to a tangible model can result in significant score improvement in beginner biology students (Höst et al., 2013), and high-quality animations of cellular processes improve scores and higher long-term memory retention among students (McClean et al., 2005). Although these and other studies have noted a positive correlation between visualization and student learning, there are still challenges to be addressed. Skeptics argue that too much visual information can lead to “cognitive overload” and thus endanger learning, although they too acknowledge that there is definite potential in visualization (Tversky et al., 2002). In fact, what can help visual data become truly effective is “interactivity”—the ability for a user to stop, start, replay, and manipulate visuals at his or her own pace. VR is an excellent platform for designing interactive and manipulatable environments.

### Interactivity: Embodied Learning in VR

VR technologies can engage learnings both cognitively and physically through immersive and interactive experiences. The theory of embodied learning posits that connecting learning events and physical actions creates a stronger impact on the individual (Kiefer and Trumpp, 2012). VR technologies can be responsive to the participants' movements in a way that activates the learners' perception of themselves as a tool for developing understanding (Stolz, 2015).

VR simulations are already widely used to develop physical skills with instruments, as a flight simulator does for a pilot's aviation skills or a surgical simulator for a doctor's surgical technique (Slater and Sanchez-Vives, 2016). VR can also help learners practice laboratory skills during virtual laboratories (Chiu et al., 2015; Lindgren et al., 2016; Jang et al., 2017). More recently, VR has been used by scientists for honing their skills in preparing molecular compounds for microscopy (Leinen et al., 2015) and for envisioning how to modify molecules to develop new pharmaceutical drugs (Cheng et al., 2012; Yuan et al., 2017; Liu et al., 2018). Scientists share computer-based visualizations with the scientific community online, drawing upon 3D models of proteins, molecules, and molecular reactions through online resources such as the Protein Databank and PyMOL (Mwalongo et al., 2016; Yuan et al., 2017). VR has enhanced the process of drug discovery by enabling scientists to investigate molecular structure and function and prompted the development of mixed reality software platforms such as Molecular Rift (Yuan et al., 2017) and Reality Convert (Borrel and Fourches, 2017). These applications of VR for science can be useful in K12 contexts by enabling learners to create embodied analogies for abstract concepts through gesture and movement (Weisberg and Newcombe, 2017). For example, a VR simulation offered higher levels of understanding and retention among high school students learning cellular biology in comparison to traditional 2D models (Tan and Waugh, 2014).

Spatial understanding is related to understanding relative size and scale, a topic many students find challenging (Jones et al., 2003). Size and scale are important to understand in science, technology, engineering, and mathematics (STEM) domains (Weisberg and Newcombe, 2017). While individuals have varying degrees of spatial understanding (Coxon et al., 2016), spatial awareness can be improved (Uttal and Cohen, 2012). Activities such as creating 3D representations of geometric shapes (Burte et al., 2017) and through gesturing while solving spatial problems (Chu and Kita, 2011) can enhance individuals' understanding. Spatial awareness is linked to perception of size and scale, which is also important in STEM topics (Jones et al., 2008). Similar to spatial awareness, understanding of size and scale can be enhanced through direct experience with objects and with distances between objects (Jones et al., 2008) and through using a body as a comparison point (Jones et al., 2009). VR has already been useful as a research tool in understanding spatial awareness (Wilson, 2013), and shows promise in developing spatial skills. VR can provide learners with virtual experience with objects and prompt learners to gesture during problem solving; both activities have the potential to improve spatial understanding and users' perception of size and scale.

Problems that require perspective taking and understanding structure are well-suited to use VR. Virtual environments can help users develop “spatial presence,” a perception of the overall VR environment and the relationships between the objects within that environment (Wirth et al., 2007). The level of embodiment achievable in VR is directly related to the level of interactivity between the user and the virtual space. 360 videos and virtual field trips are already being used in classrooms to help students learn geography and cultural awareness due to the lower cost of the equipment, however, the user has limited ability to interact with the experience(Brown and Green, 2016; Korbey, 2017; Minocha et al., 2017). Interactive simulations and virtual laboratories have helped students understand electrostatics and forces in physics (Salzman et al., 1999; Pirker et al., 2013), and mathematics (Mizell et al., 2002; Guerrero et al., 2015). Laboratories and simulations require more resources to design than virtual field trips, but the additional interaction supports a deeper level of embodied learning (Potkonjak et al., 2016; Jang et al., 2017).

### Collaborative: Learning in VR

The movement from room scale CAVE Automatic Virtual Environment (CAVE) to head-mounted displays (HMD) has decreased the cost of VR, yet these technologies have focused heavily on the individual's experience (Hew and Cheung, 2010; Slater and Sanchez-Vives, 2016). As technology and connectivity improves, VR will include collaboration between individuals in HMDs, requiring a new understanding of how technology can enable new forms of communication between individuals (Gugenheimer et al., 2017). Designers must balance the users' attention to their own experience and explore how to create a sense of shared presence, or co-presence, in the virtual world (Campos-Castillo, 2012).

Principles of collaborative learning such as interdependence, thoughtful formation of groups, individual accountability, and attention to social skill development are also useful considerations in VR environments (Cuseo, 1997; Lee, 2009). Activities that require individuals to work together in order to accomplish goals create what Johnson and Johnson (1989) call “positive interdependence” among team members; the structure of the activity necessitates a joint effort. Since virtual environments are still relatively novel, both rules and roles can be useful in structuring collaboration. Jensen and Konradsen (2018) used games as a way to create rules for social interaction and roles for individuals in virtual problem-based activities. Roles also helped visitors engage with a VR museum exhibit experience on an aircraft carrier (Zhou et al., 2016). Middle school students in the EvoRoom VR environment EvoRoom environment benefited from clear roles in gathering and sharing information with their peers (Lui and Slotta, 2014).

In addition to clear roles, a range of expertise helps foster interdependence in virtual teams (Weber and Kim, 2015). One way to establish roles is to structure distributed teamwork through roles and access to different forms of technology and information (text based, 2D, 3D, VR) that must be synthesized to solve a problem. This redistribution can create power dynamics within the group. In comparing virtual to in person problem solving among teams of people using 2D, 3D, and VR interfaces, (Slater et al., 2000; Slater and Sanchez-Vives, 2016) found that the individual in VR was more likely to emerge as the leader, even if that same person did not take on a leadership role in the in-person project. Spante et al. (2006) also studied puzzle solving across VR and 2D systems; they found that team members assumed both had the same view until they traded places. Having different viewpoints enhanced collaboration, creating what Spante et al. (2006) termed “the good inequality”. Gugenheimer et al. (2017) created a system where individuals in HMD could interact with individuals outside of VR through a “FaceDisplay,” a touch screen interface. Teamwork can be reinforced by structuring environments to providing team members with complementary information and different views of information; furthermore, students also gain appreciation of how different forms of media may be more appropriate for understanding certain concepts.

## An Example in Progress Cellverse—A Collaborative Learning Environment in Virtual Reality (CLEVR)

**Figure 1:**
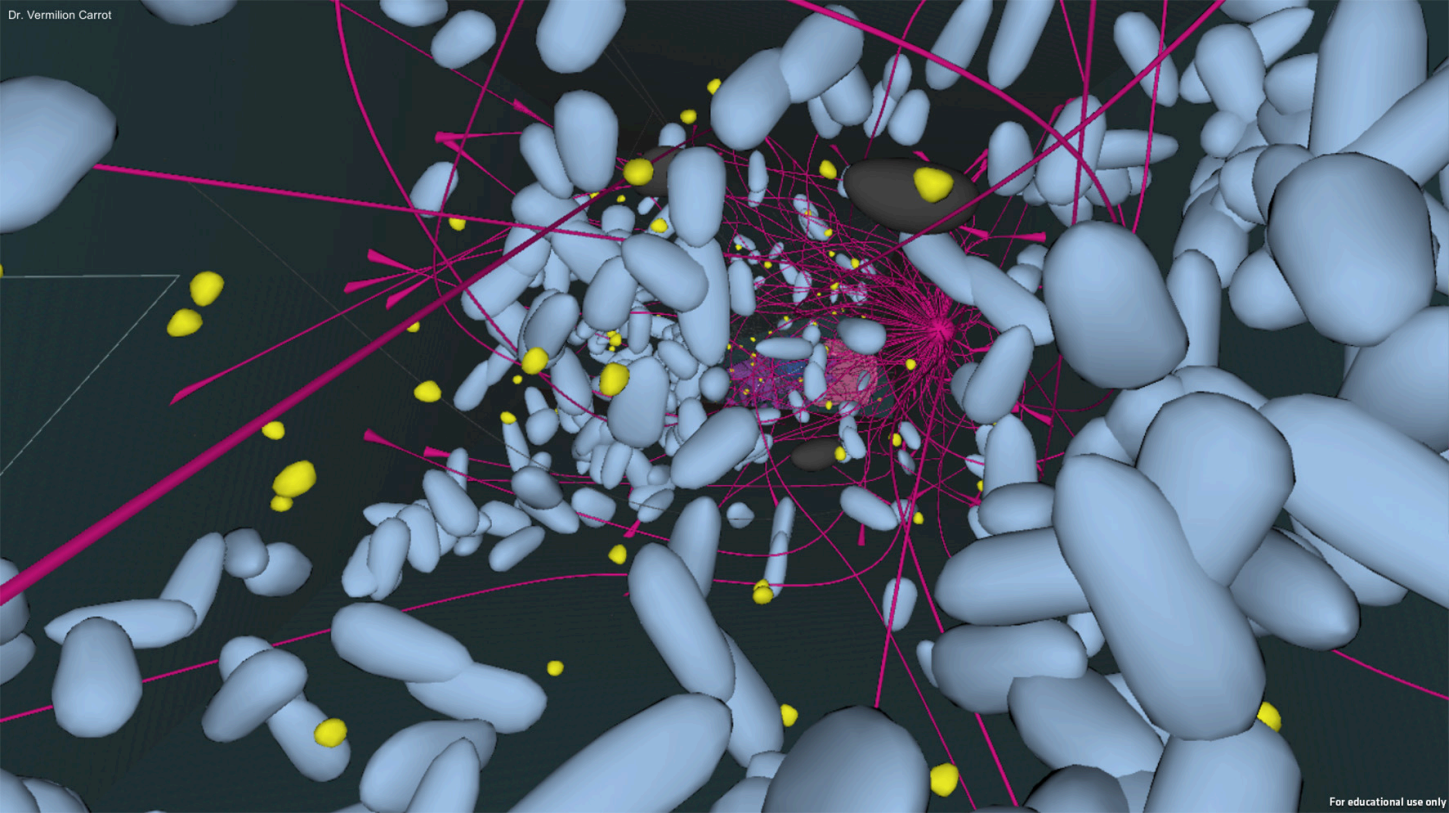
An example of the Explorer view inside the cell in Cellverse.

**Figure 2:**
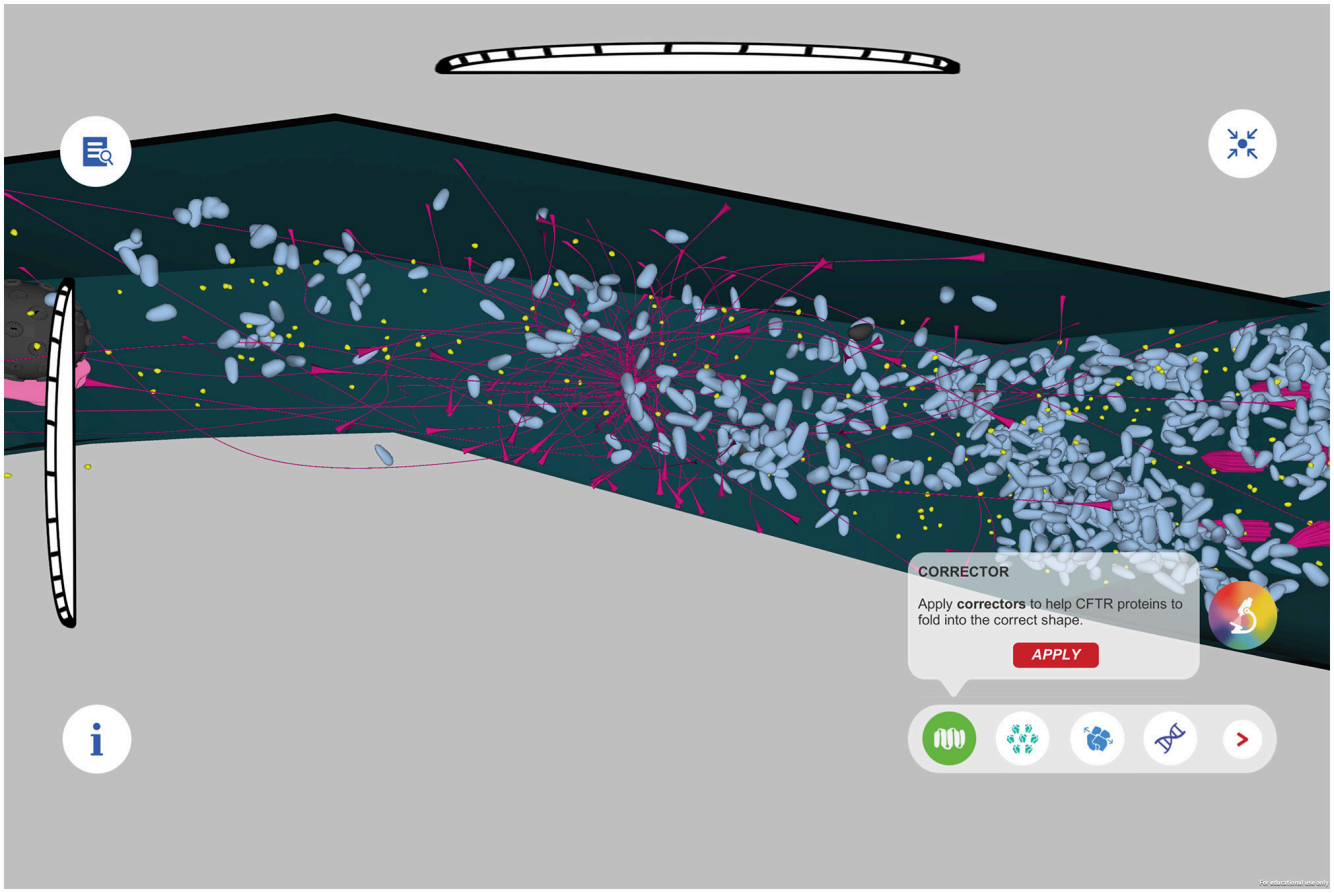
An example of the Navigator view outside the cell in Cellverse.

We now apply some of these ideas about embodiment and collaborative learning to a game-based learning project currently under development, Cellverse. In Cellverse students learn about cells and the process of converting DNA to proteins through an interactive problem-based game. Working in small teams of two or three, students examine a living cell from within. The Explorer wears a head mounted display and moves through the cell in VR to observe function and structure, as shown in Figure 1. The Navigator uses a tablet-based toolkit of disease descriptions, stains, tags, and measurement devices to gather data and focus the visualizations using a table, as shown in Figure 2. The experience is being designed with a distribution of data available for players in a way that students must communicate to solve the puzzle together.

A central question is—why use VR? Virtual reality allows students to experience the cellular environment as an active explorer, rather than a passive observer. It also gives students an appreciation for the density of the cell, the size and scale of organelles relative to each other and to other molecules in the cell, spatial relationships between the organelles, and the cellular structure. Structure and spatial orientation are both important in understanding the central dogma, when DNA is first transcribed to mRNA and translated by tRNA into long amino acid chains that become proteins.

We draw connections between the effective practices we have found in in the literature using the affordances of VR and our intentions for this project.

### Authentic

We situate student learning in the context of biology, both in the game narrative and the game environment. We have built a cellular environment that matches current research on cells, with ongoing input and feedback from cellular biologists and other cell biology experts. Whenever possible, we have incorporated tools and activities that scientists would use as in-game functions. For example, students can highlight specific organelles and structures within the virtual environment using simultaneous label-free autofluorescence-multiharmonic (SLAM) microscopy (You et al., 2018). Cells are densely packed, which is challenging to render and can be overwhelming to users. We continually balance how to represent the cell most authentically while maintaining presence within the experience and minimizing cognitive load.

### Interactive

The game-based format provides a high degree of interaction between students and the concepts included in the VR environment, which has been linked to deeper learning (Lindgren et al., 2016; Jang et al., 2017). The game-based format also provides ongoing feedback to the players, which also assists the learning process (Merchant et al., 2012). Through the game, we aim to transform a topic that is often passive and vocabulary-based into an active, embodied experience.

We also incorporate aspects of biology within the game narrative and the game environment. We are building a cellular environment that matches current research on cells, with ongoing input and feedback from cellular biologists. Students will learn what a cell biologist might do by using tools such as (SLAM) microscopy (You et al., 2018).

### Collaborative

We are building interdependence among team members into the design of the project by creating rules, establishing roles, and distributing resources between players. Rules are established before students take on roles in the game; either as explorers or navigators. The explorer will see the 3D VR view of the world and will complete tasks that involve spatial relationships between organelles, identifying protein structures, and tracking processes within the cell. The navigator has access to information on 2D and 3D flat screen models to help guide the explorer and to work with other team members as they identify organelles, proteins, and even DNA and RNA sequences that could provide helpful clues in the game. We plan to build different levels in the game to allow students to rotate through team roles.

Collaborative activities can be enhanced through different modalities. We implemented a number of functions that would allow users to communicate to each other across platforms, including but not limited to “light beacons” that can be placed within the virtual environment and functions such as SLAM microscopy that reflect how real-world scientists mark organelles. These functions do not only allow users to communicate with each other through non-verbal manners, but also enhance their collaborative experience and create embodied learning within the virtual environment.

## Challenges

There have been a number of challenges that we have confronted while building and implementing Cellverse. As Cellverse is a complex environment with many moving parts, users risk becoming nauseated if there is too much activity, or not enough computer processor power to render the activity in real time. A high frame rate, thus, is vital for a smooth VR experience; too much detail or too many objects within the virtual world can reduce the frame rate and cause nausea (Jerald, 2015). We have had to compromise authenticity with playability, and reduced the details of certain structures in order to maintain a comfortable frame rate.

Creating a balanced flow of information between the two players has been challenging. Effective and worthwhile collaboration happens when each player is equally involved and are able to fill in whatever information their partner does not possess. We have explored different ways to foster collaboration through distribution of information resources to the players.

While our goal is to create an authentic environment, scientific understanding is continually advancing. We have also had to make choices about the specificity of our cellular model and the number of processes we can represent in a realistic design timeframe. We have also noted that in an ever-evolving field like microbiology, application authenticity in educational material remains a challenge. There are new discoveries made regarding cells and cell structure every day, and it is in our best interest to make Cellverse as accurate to these discoveries as possible. However, it sometimes means that we do have to change aspects of the game that may not be immediately noticeable to student players. Although they may not be consciously aware of these changes, it is our belief that making the Cellverse environment authentic will allow students to come away with a more well-rounded understanding of cells.

## Feasibility

We are also attuned to how CLEVR could be integrated into curricula and implemented in classrooms. Our partner teachers have confirmed that the cell and central dogma are important topics in introductory biology. Teachers are helping us imagine how to incorporate VR technology in a feasible way in today's classrooms, and also provide insight into design features of the game that can help the game run smoothly. For example, while students may be excited to try the 3D VR experience, having all students in the class in headsets simultaneously may be a challenge. Conversely, some students may not want to wear headsets, or may be absent from school during the activities, so the activity should be designed so that team members can take on different roles and responsibilities if not all members are there.

Despite these opportunities, the cost of developing quality educational materials remains relatively high. Although the cost of VR has decreased over the years (Korbey, 2017), investing in VR requires significant resources. The labor involved in creating immersive, interactive, and accurate educational VR material is also great. It is then necessary to capitalize on all possible affordances of VR, and to carefully allocate resources so that more individuals can participate—perhaps at once—and benefit from the experience in the long term.

## Conclusion

Now that VR technology is within reach of educational settings, learning designers and educators can focus on how best to incorporate VR into educational contexts. In this article, we discuss and provide an example of how VR experiences can represent authentic contexts, focus on embodied experiences, and how to structure the environment to foster teamwork and collaboration by having participants view and synthesize different types of data across immersive and non-immersive formats. These parameters can be used to develop effective and engaging learning environments based on our current understanding of VR in education. When moving forward, researchers, developers, and educators should investigate how each of these factors can be fashioned to optimize learning, identify affordances and challenges that may emerge as VR becomes more widespread, and incorporate findings and feedback into future development.

## Ethics Statement

This study was carried out in accordance with the The Common Rule, 45 CFR pt. 46, with informed written consent from all subjects. All subjects gave written consent in accordance with the Declaration of Helsinki. The protocol was approved by the Committee on the use of Humans as Experimental Subjects (COUHES) at MIT (#10707095354).

## Author Contributions

MT did the planning for the paper, read and synthesized literature notes, and wrote the paper. AW read and compiled paper notes, wrote the section on students' understanding of cells, and helped review drafts of the manuscript. DR helped review the paper from a game designer perspective and edited the paper. EK advised MT as she formulated the paper idea, helped imagine the direction for the paper, and reviewed and edited the paper multiple times, providing indepth feedback.

### Conflict of Interest Statement

The authors declare that the research was conducted in the absence of any commercial or financial relationships that could be construed as a potential conflict of interest.
